# A Virtual Kitchen Protocol to Measure Everyday Memory Functioning for Meal Preparation

**DOI:** 10.3390/brainsci11050571

**Published:** 2021-04-29

**Authors:** Michael D. Barnett, Lucas G. Childers, Thomas D. Parsons

**Affiliations:** 1 Department of Psychology and Counseling, The University of Texas at Tyler, 3900 University Boulevard, Tyler, TX 75799, USA; lchilders2@patriots.uttyler.edu; 2 iCenter for Affective Neurotechnologies (iCAN), College of Information, University of North Texas, Denton, TX 76203, USA; thomas.parsons@unt.edu

**Keywords:** activities of daily living, instrumental activities of daily living, computer use, older adults, verbal memory, visual memory, video games, serious games

## Abstract

In this study, we developed the Virtual Kitchen Protocol (VKP), a virtual reality-based measure of everyday memory functioning for meal preparation tasks. We investigated the construct validity of the VKP by comparing the performance of young adults (*n* = 41) and older adults without (*n* = 52) and with (*n* = 7) a neurocognitive diagnosis, as well as by examining correlations with standardized measures of verbal and visual memory. The results show that young adults had higher recall than older adults and that the VKP was sensitive to neurocognitive impairment among older adults. The VKP demonstrated moderate to high correlations with other memory tests. These results support the construct validity of the VKP and suggest that it holds promise as a virtual reality-based measure of memory for meal preparation tasks.

## 1. Introduction

### 1.1. A Virtual Kitchen Protocol to Measure Everyday Memory Functioning

Traditional neuropsychological tests use abstract experimentally derived theoretical constructs to uncover the behavioral consequences of brain damage [1,2]. While such tests have been successful, they may offer limited insight into individuals’ everyday functioning [3,4]. As an alternative, function-led tests of everyday abilities [5] using novel simulations offer a means of measuring everyday abilities by simulating tasks within standardized environments [6,7,8,9]. These high dimensional (didactive, integrative, and multi-dimensional) approaches place patients in realistic environments that may increase the ecological validity of testing and practice of daily life skills [10]. Likewise, virtual environments integrate controllable simulations for assessing the patient’s functional capacities [11]. One important everyday task—indeed, an instrumental task of daily living [9,12]—is meal preparation. The purpose of this study was to investigate the use of a simulated meal preparation environment for assessing everyday memory functioning. In order to do so, we developed the Virtual Kitchen Protocol and investigated its construct validity.

### 1.2. Meal Preparation Tasks

As Burgess et al. (2006) argued, we know very little about how the brain organizes simple everyday activities like cooking. Cooking appears to involve a variety of skills, such as the intelligent use of space [13], prospective and working memory [12,14], as well as processing speed, visual perceptual reasoning, and unconscious tracking [13,15,16,17,18]. Memory and recall of the steps needed to prepare specific meals may also share much in common with other memory tasks. For example, simpler task demands and more frequent practice are associated with better performance [13].

The limited extant research on meal preparation has primarily relied upon in vivo meal preparation tasks; however, these involve inherent limitations for both clinical and research applications [19,20]. At a practical level, a real-life kitchen requires the dedication of large and costly spaces with specialized equipment (e.g., kitchen appliances) that otherwise would not generally be found in clinical or research environments. Additionally, in vivo meal preparation tasks often involve dangerous activities such as cutting and heating food, introducing issues of safety. These problems lead to measurement problems such as standardization—it is difficult to recreate the exact kitchen environment in multiple clinics [21,22]. Virtual reality offers the opportunity to simulate meal preparation tasks in a way that is safe, standardized, and scalable.

We designed and developed the Virtual Kitchen Protocol (The Virtual Kitchen Protocol is available online as supplemental material: https://mfr.osf.io/render?url=https%3A%2F%2Fosf.io%2Fac2fx%2Fdownload (accessed on 26 March 2021)) to measure memory for meal preparation tasks in virtual reality. The VKP has participants prepare six small meals that vary in their complexity. We wanted multiple meals in order to capture sufficient variation in performance. However, from the perspective of measuring neuropsychological abilities, there are a number of potentially confounding variables and difficulties. Different meals may involve different amounts of time. For example, it may take considerably less time to cook an egg than to cook a steak, and having an individual wait long durations while an item cooks may, although realistic, lead to wasted time. We were primarily interested in individuals’ ability to learn and remember both the items and the steps to produce them. Hence, we chose a platform that simulated meal preparation (i.e., cooking) without excessive delays due to cooking times.

### 1.3. The Current Study

Memory researchers often distinguish between procedural memory (implicit memory for motor skills) and episodic memory (explicit memory of recalled events; [23]). Lost in this distinction is the notion that individuals often must learn and remember complex actions in an explicit manner, relying on their conscious recall of fine-tuned details that aid and assist their performance with the task at hand. In this study, we developed a virtual reality-based measure of everyday memory functioning: the Virtual Kitchen Protocol (VKP). The purpose of this study was to investigate the construct validity of the VKP by investigating correlations with the California Verbal Learning Test–II (CVLT-II) and Wechsler Memory Scale–IV Visual Reproduction (WMS-IV VR) among young adults and older adults with and without a neurocognitive diagnosis. Previous research found that young adults outperform older adults on episodic memory tasks [24,25], including those in virtual reality [26,27]. Therefore, we hypothesized (*H*_1_) that, compared to older adults, young adults would have better memory for meal preparation tasks (i.e., VKP-short delay recall, VKP-long delay recall, and VKP-recognition). We also hypothesized (*H*_2_) that VKP scores would demonstrate moderate to high correlations with analogous variables on the CVLT-II (i.e., VKP-short delay recall with CVLT-II SDFR and VR I; VKP-long delay recall with CVLT-II LDFR and VR II; and recognition tasks across all three tests). Finally, because men and women may differ in their experience with cooking in real-world environments and other household chores as defined by conventional gender roles [28], we considered possible confounds such as gender, comfort with cooking, cooking frequency, comfort with computers, and computer use frequency.

## 2. Materials and Methods

### 2.1. Participants

Participants consisted of young adults, older adults without a neurocognitive diagnosis, and older adults with a neurocognitive diagnosis. The young adult sample (*n* = 41; 65.9% female, 31.7% male, 2.4% other/missing; age 18–26, *M* = 18.85, *SD* = 1.57) consisted of college students enrolled in a psychology course at a public university in the southern U.S. These students were recruited from the departments’ SONA system, a website where students can volunteer to participate in research studies in exchange for course credit with an alternative assignment available. Regarding race/ethnicity, 82.9% of the students identified as white/Caucasian, 4.9% as Pacific Islander, 2.4% as black/African American, and 9.7% as other/missing. The sample of older adults without a neurocognitive diagnosis (*n* = 52, 53.8% female, 40.4% male, 5.8% other/missing; age 58–89, *M* = 72.71, *SD* = 7.45) were recruited from senior living centers, civic organizations, and other community groups to participate in a research project. Regarding race/ethnicity, 88.5% identified as white/Caucasian, 1.9% as black/African American, and 9.6% as other/missing. The sample of older adults with a neurocognitive diagnosis (*n* = 7; 57.1% male, 42.9% female, age 60–90, *M* = 78.00, *SD* = 9.17) were patients referred for a neuropsychological evaluation by the local Alzheimer’s Alliance organization and other area health care providers. Five of these patients were diagnosed with a major neurocognitive disorder (1 due to normal pressure hydrocephalus, 1 due to vascular dementia, and 3 due to unspecified causes), and one patient with a mild neurocognitive disorder due to vascular dementia. Regarding race/ethnicity, 6 of these participants identified as white/Caucasian, and 1 identified as Native American. All testing took place in the same location, a university-affiliated neuropsychological testing center. Participants were screened for photosensitivity, epilepsy, or other medical conditions that might post a safety concern.

### 2.2. Measures

**Estimated Premorbid Intelligence.** The Wechsler Test of Adult Reading (WTAR; [29]) is a widely used neuropsychological test used to measure and estimate the premorbid intellectual functioning of participants. The WTAR is a conventional pen-and-paper neuropsychological test assessing participants verbal abilities through reading a series of 50 words looking for atypical grapheme to phoneme relationships. Standard scores were used.

**Comfort with Computers and Computer Use Frequency.** Two items measured comfort with computers. The first item was: “*I have avoided computers because they are unfamiliar and somewhat intimidating to me*,” and participants responded on a 5-point Likert scale. The second item was, “*How would you rate your computer competency in terms of knowing how to use a computer?*” and participants responded on a Likert-type scale ranging from 1 = *completely inexperienced* to 5 = *very experienced.* These two items, which were highly correlated (*r* = 0.71, *p* < 0.001), were summed to form comfort with computers. Frequency of computer use was measured by asking participants how many hours per week on average they spend using a computer for 8 tasks: *word processing, programming, playing games, data entry/processing, graphic design/art, surfing the internet, emailing,* and *other* and summing these.

**Comfort with Cooking and Cooking Frequency.** Comfort with cooking was measured with the item “*How comfortable are you cooking meals in a real kitchen?*”, to which participants responded on a Likert-type scale ranging from 1 = *not at all comfortable* to 5 = *very comfortable.* Cooking frequency was measured with the item “*How often to you prepare cooked meals in a real kitchen?*”, to which participants responded on a Likert-type scale ranging from 1 = *never* to 5 = *very frequently*.

**Figure 1:**
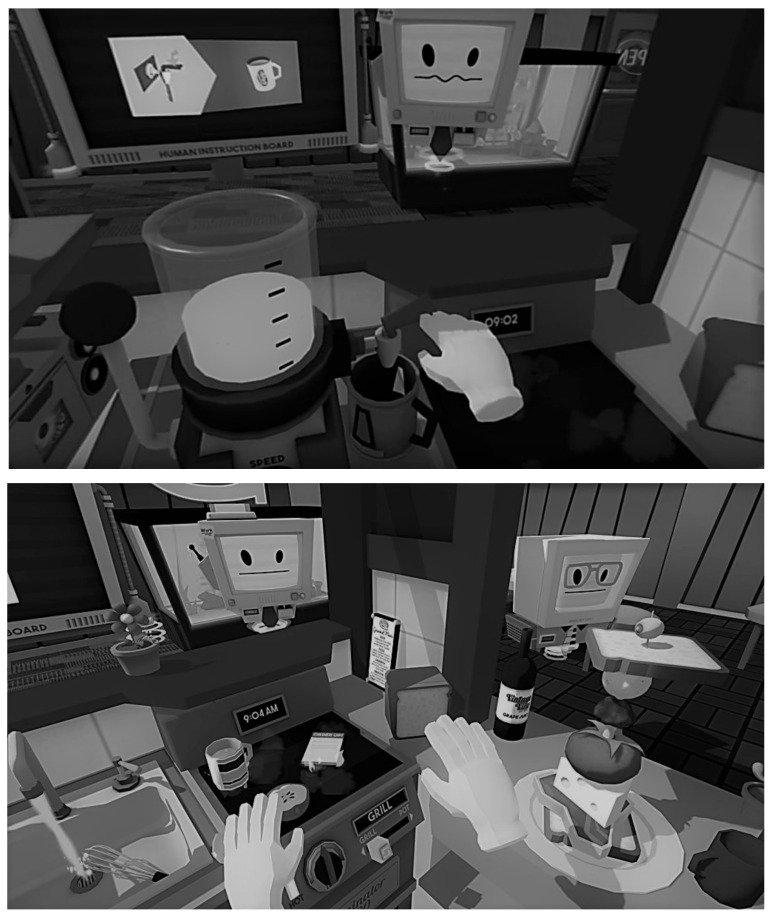
Grayscale images of Job Simulator for the HTC Vive©. Copyright © 2020 by Owlchemy Labs.

**Virtual Kitchen Protocol: Memory.** The Virtual Kitchen Protocol (VKP) is a virtual reality-based measure of everyday memory for meal preparation. The VKP uses Job Simulator [30], a commercially available virtual reality application (Copyright © 2021 by Owlchemy Labs), shown in Figure 1.

The VKP involves making 18 meals in a virtual reality environment. Individuals first go through a tutorial introducing them to the virtual reality environment and controls. Next, they go through a teaching trial to orient themselves to the task and then get six trials to make six meals. Immediately following this, individuals then go through a short delay recall trial and then get six trials to make six meals. The long delay recall takes place after 20 min. The recognition task, which consists of 16 forced-choice items, then follows.

**Visual Memory.** The Wechsler Memory Scale–IV (WMS-IV; [31]) is a widely used neuropsychological assessment measuring auditory and visual memory capabilities. Specifically, the Visual Reproduction (VR) section of the WMS assesses patient’s ability to encode nonverbal visual stimuli and recall in both immediate, delayed, and forced choice memory of said stimuli.

**Verbal Memory.** The California Verbal Learning Test–II (CVLT-II; [32]) is a widely used neuropsychological assessment measuring auditory learning capabilities and memory deficits. The CVLT assesses immediate and delayed free recall, short delay and long delayed cued recall, and long delayed force choice recall.

**Materials.** Participants played Job Simulator: The 2050 Archives (Copyright © 2016) by Owlchemy Labs on the HTC Vive ©, which features dual AMOLED 3.6” diagonal screens with a resolution of 1080 × 1200 pixels per eye, a refresh rate of 90 Hz and a 110° field of view. The minimum system requirements for Job Simulator were: an operating system of Windows^®^ 7 SP1 or better, processor of Intel^®^ i5-4590 equivalent or better, memory of 4GB RAM, graphics card of NVIDIA^®^ GeForce^TM^ GTX 970/AMD^®^ Radeon^TM^ R9 290 equivalent or better, and storage of 1GB. This was run on an Alienware Aurora R8 computer, which features an Intel^®^ Core^TM^ i9-9900k CPU (8-Core/16-Thread, 16MB Cache, 3.60 GHz processor, overclocked up to 4.7 GHz across all cores), NVIDIA^®^ GeForce^TM^ RTX 2080 Ti OC with 11GB GDDR6 video card, and 64GB DDR4 XMP at 2933MHz HyperX RAM on a 64-bit operating system, with an x64-based processor.

### 2.3. Procedures

This study was approved by the university’s committee for the protection of human subjects. Informed consent was obtained from all participants. Participants completed the VKP on an HTC Vive HMD with one controller for each hand. For safety, participants completed the VKP while in a seated position.

## 3. Results

The Virtual Kitchen Protocol (VKP) variables demonstrated negative skew (short delay recall: −1.18; long delayed recall: −1.38, recognition: −4.83) that was not improved through linear transformation; therefore, we used the raw scores in all analyses. We used a one-way MANCOVA to compare three groups (i.e., young adults (YA), older adults without a neurocognitive diagnosis (OA_NoDx_), and those with a neurocognitive diagnosis (OA_Dx_)) on the three VKP variables (i.e., short delay recall (SDR), long delay recall (LDR), and recognition (Rec)) while controlling for the effects of gender (dummy coded 0 = female, 1 = male), estimated premorbid intelligence (i.e., WTAR SS), comfort with cooking, cooking frequency, comfort with computers, and computer use frequency. There was a large multivariate difference between these three groups on the VKP variables, *F*(6178) = 24.72, Wilks’ *λ* = 0.30, *p* < 0.001, *η_p_^2^* = 0.45, observed power = 1.00. Large univariate differences, displayed in Table 1, were found between these three groups. Tukey’s HSD post hoc found all group differences significant at *p* < 0.001 for VKP short delay recall and VKP long delay recall, with young adults scoring the highest, followed by older adults without a neurocognitive diagnosis, followed by older adults with a neurocognitive diagnosis. On VKP recognition, however, older adults with a neurocognitive diagnosis scored significantly lower than the other two groups (at *p* < 0.001), but the difference between young adults and older adults without a neurocognitive diagnosis was not significant (*p* = 0.53).

At the multivariate level, gender was the only significant covariate, *F*(3,89) = 3.41, Wilks’ *λ* = 0.90, *p =* 0.02, *η_p_^2^* = 0.10; however, this effect size was small (*η_p_^2^* = 0.10) and with insufficient power (.75). At the univariate level, gender demonstrated a significant effect only on VKP short delay recall, *F*(1,91) = 6.59, *p* = 0.01; again, this effect size was small (*η_p_^2^* = 0.07) and power was insufficient (.72). At the univariate level, estimated premorbid intelligence had a significant effect on VKP recognition, *F*(1,91) = 5.57, *p* = 0.02. However, again, this effect size was small (*η_p_^2^* = 0.06) and power was insufficient (.65). Comfort with computers had a significant effect on VKP short delay recall, *F*(1,91) = 4.97, *p* = 0.03; again, the effect size was small (*η_p_^2^* = 0.05) and power was lacking (.60).

Bivariate correlations between and descriptive statistics for all variables of interest are displayed in Table 2. VKP short delay recall was highly correlated with the CVLT-II short delay free recall (*r* = 0.73, *p* < 0.001), and likewise for long delay (*r* = 0.70, *p* < 0.001), and recognition (*r* = 0.77, *p* < 0.001). VKP variables demonstrated more modest correlations with WMS-IV visual reproduction scores, with a medium correlation between VKP short delay recall and VR I (*r* = 0.30, *p* = 0.003), a large correlation between VKP long delay recall and VR II (*r* = 0.64, *p* < 0.001), and a medium correlation between VKP recognition and VR II recognition (*r* = 0.25, *p* = 0.012). Next, we partialled out variance for age cohort, neurocognitive diagnosis, gender, estimated premorbid intelligence, comfort with cooking, cooking frequency, comfort with computers, and computer use frequency; relationships between VKP variables and analogous CVLT-II variables remained statistically significant, but relationships with WMS-IV variables did not.

## 4. Discussion

In this study, we introduced the Virtual Kitchen Protocol (VKP), a virtual reality-based measure of everyday memory functioning for meal preparation, and investigated its construct validity. The results are generally consistent with *H*_1_, in that young adults obtained higher scores on the VKP recall than older adults without a neurocognitive diagnosis, who in turn obtained higher scores than older adults with a neurocognitive diagnosis. These results are consistent with a large body of literature showing that young adults outperform older adults on measures of episodic memory, as measured both by traditional neuropsychological tests [24] as well as virtual reality-based tests [14,26,27]. Older adults with a neurocognitive diagnosis scored lower on VKP recognition than the other two groups, suggesting that the forced-choice recognition task may be sensitive to cognitive impairment. However, young adults and older adults without a neurocognitive diagnosis had similar scores on VKP recognition. This may represent a ceiling effect for VKP recognition. This was reflected in the negative skew of the VKP variables, particularly the forced-choice recognition task. However, again, the level of skew was consistent with that seen on the CVLT-II and WMS-IV VR, and negative skew is a common feature of recall tests and particularly of recognition tests.

The results also generally support *H*_2_. The VKP memory measures demonstrated correlations with their verbal memory counterparts on the CVLT-II. This pattern of results remained even when controlling for possible confounding variables (age cohort, neurocognitive diagnosis, gender, estimated premorbid intelligence, comfort with cooking, cooking frequency, comfort with computers, and computer use frequency). Correlations between VKP scales and their visual memory counterparts (WMS-IV VR I and II) were somewhat more moderate, and these relationships were not significant after controls were applied. The findings that performance was more strongly correlated with verbal memory than visual memory are consistent with other VR-based measures of everyday memory abilities [27].

Given that women may have carried out more meal preparation than men [28], we investigated possible differences by gender. There was evidence of some age, gender, and age by gender interactions on the VKP variables, particularly VKP short delay recall. Interestingly, young adult men scored the highest followed by young adult women, older adult women, and finally older adult men. Thus, although older adult women outperformed their male counterparts, young adult men outperformed their female counterparts. This may reflect a gender effect in which men may outperform women on certain visuo-spatial tasks [33,34]. Another explanation could be that prior exposure to videogames would have made a difference between young men and women [35]. Indeed, results of post hoc analyses found that the interaction between gender and cohort was no longer significant when computer use and perceived computer competency were added in the model, although the age cohort and gender effects were still significant. Taken together, the results suggest that gender may have some effect on the VKP short delay recall.

It is important to note that this study was limited in several ways. The samples were fairly small in size and may not be fully representative of their respective populations. The young adult sample consisted of college students and, although we controlled for estimated intelligence, the older adult samples were not matched according to education. Additionally, all three samples were drawn from the same geographic area and not very diverse with regard to race/ethnicity. The data are also cross-sectional in nature, and although this is adequate to highlight patterns of shared variance, it is not enough to conclude any predictive capabilities of the VKP. Finally, while this study suggests that the VKP may have utility as a measure of everyday memory for meal preparation, performance on such a task cannot necessarily be generalized to other instrumental activities of daily living.

## 5. Conclusions

Researchers have discussed the notion that video games may have applications beyond entertainment (serious games; [36]). In this study, we introduced a serious game: the VKP. The results of this study provide initial support for the construct validity of the VKP as a measure of everyday memory in the area of meal preparation. This study also suggests a number of directions for future work with the VKP. Previous researchers pointed out that it was not known which abilities were involved in meal preparation [3,5]. The results of this study are consistent with the notion that meal preparation involves memory; however, in vivo studies suggest the additional involvement of other cognitive abilities such as processing speed, visual perceptual reasoning, and unconscious tracking have been observed in complex tasks like cooking [13,15,16,17,18]. Thus, future research could investigate relationships between VKP variables and other neuropsychological domains (e.g., fluid intelligence, attention, processing speed, and executive functioning) as well as with instrumental activities of daily living and performance-based functional abilities. With additional research support, the VKP may eventually have clinical applications as a measure of everyday memory and functional abilities since the ability to prepare meals is often an important consideration in whether individuals should live independently. By using a commercially available VR video game, the VKP could be implemented as a serious game at fairly low cost and without great computer expertise.

## Figures and Tables

**Table 1:** Univariate group differences and descriptive statistics on Virtual Kitchen Protocol variables (*n* = 100).

	*F* (2,91)	*η_p_^2^*	*M* (*SD*)	*M* (*SD*)	*M* (*SD*)
			YA (*n* = 41)	OA_NoDx_ (*n* = 52)	OA_Dx_ (*n* = 7)
VKP SDR	39.85 *	0.47	58.44 (6.82)	42.15 (15.63)	13.27 (18.54)
VKP LDR	72.11 *	0.61	60.83 (5.78)	44.88 (10.82)	15.71 (18.52)
VKP Rec	29.70 *	0.38	15.83 (.38)	15.65 (.55)	12.86 (3.34)

* *p* < 0.001. VKP = Virtual Kitchen Protocol. SDR = Short Delay Recall. LDR = Long Delay Recall. Rec = Recognition.

**Table 2:** Bivariate correlations between and descriptive statistics for all variables of interest among all participants (*n* = 100).

	1	2	3	4	5	6	7	8	9
1. CVLT SDFR	-	0.88 ***	0.39 ***	0.31 **	0.54 ***	0.28 **	0.73 *** (0.48 ***)	0.70 *** (0.42 ***)	0.44 *** (0.11)
2. CVLT LDFR		-	0.40 ***	0.33 **	0.52 ***	0.29 **	0.74 *** (0.52 ***)	0.70 *** (0.43 ***)	0.49 *** (0.18)
3. CVLT Rec			-	0.15	0.24 *	0.18	0.39 *** (0.19)	0.44 *** (0.22 *)	0.77 *** (0.67 ***)
4. WMS VR1				-	0.35 ***	0.16	0.30 ** (0.03)	0.32 ** (−0.02)	0.21 * (0.05)
5. WMS VR2					-	0.32 **	0.63 *** (0.25 *)	0.64 *** (0.19)	0.36 *** (0.00)
6. WMS Rec						-	0.27 ** (0.04)	0.27 ** (0.02)	0.25 * (0.16)
7. VKP SDR							-	0.83 *** (0.63 ***)	0.45 *** (0.12)
8. VKP LDR								-	0.55 *** (0.24 *)
9. VKP Rec									-
*M*	10.53	10.70	99.12	36.17	25.38	6.28	46.81	49.38	15.53
*SD*	3.81	3.86	5.00	12.67	10.96	4.61	17.68	15.48	1.20

CVLT = CVLT-2, SDFR = short delay free recall, LDFR = long delay free recall, Rec = recognition. WMS VR = WMS-IV visual reproduction. VKP = Virtual Kitchen Protocol, SDR = short delay recall, LDR = long delay recall. Numbers in parentheses are partial correlations (*df* = 92) controlling for dummy-coded gender, age cohort, and neurocognitive diagnosis as well as estimated premorbid intelligence, comfort with cooking, cooking frequency, comfort with computers, and computer use frequency. * *p* < 0.05, ** *p* < 0.01, *** *p* < 0.001.
